# Timely germline *BRCA* testing after invasive breast cancer promotes contralateral risk-reducing mastectomy and improves survival: an observational retrospective study

**DOI:** 10.1007/s10549-025-07726-2

**Published:** 2025-05-23

**Authors:** Aleksandar M. Kostov, Maj-Britt Jensen, Bent Ejlertsen, Mads Thomassen, Maria Rossing, Inge S. Pedersen, Annabeth H. Petersen, Lise Lotte Christensen, Karin A. W. Wadt, Luis C. Berrocal-Almanza, Miguel Miranda, Anne-Vibeke Lænkholm

**Affiliations:** 1 Department of Surgical Pathology, Zealand University Hospital, Sygehusvej 9-11, 4000 Roskilde, Denmark; 2 Danish Breast Cancer Cooperative Group, Department of Oncology, Rigshospitalet, Copenhagen University Hospital, Copenhagen, Denmark; 3 Department of Clinical Medicine, Faculty of Health and Medical Sciences, University of Copenhagen, Copenhagen, Denmark; 4 Department of Clinical Genetics, Odense University Hospital, Odense, Denmark; 5 Human Genetics, Department of Clinical Research, University of Southern Denmark, Odense, Denmark; 6 Clinical Genome Center, University of Southern Denmark and Region of Southern Denmark, Odense, Denmark; 7 Department of Genomic Medicine, Rigshospitalet, Copenhagen University Hospital, Copenhagen, Denmark; 8 Department of Clinical Medicine, Aalborg University, Aalborg, Denmark; 9 Department of Molecular Diagnostics, Aalborg University Hospital, Aalborg, Denmark; 10 Clinical Cancer Research Center, Aalborg University Hospital, Aalborg, Denmark; 11 Department of Clinical Genetics, Vejle Hospital, University Hospital of Southern Denmark, Vejle, Denmark; 12 Department of Molecular Medicine (MOMA), Aarhus University Hospital, Aarhus, Denmark; 13 Department of Clinical Genetics, Rigshospitalet, Copenhagen University Hospital, Copenhagen, Denmark; 14 Oncology Outcomes Research, AstraZeneca, Cambridge, UK

**Keywords:** Genetic screening, Hereditary breast cancer, Danish breast cancer cooperative group, Risk-reducing surgery, Contralateral mastectomy

## Abstract

**Purpose:**

To report the rates of risk-reducing surgery (RRS) following germline testing for *BRCA1/2* (likely) pathogenic variants (*BRCApv*) and to assess the impact of RRS and *BRCA* status on survival after surgical treatment for unilateral breast cancer (BC).

**Methods:**

We identified 7145 women with BC (2000–2017), a *BRCA* test and median follow-up of 10.8 years from the Danish Breast Cancer Cooperative Group’s clinical database. Distant recurrence-free (DRFS) and overall survival (OS) according to *BRCA* status were evaluated using the Kaplan–Meier method. Hazard ratios (HR) for *BRCApv vs. BRCA wild-type*, contralateral risk-reducing mastectomy (CRRM), and risk-reducing bilateral salpingo-oophorectomy (RRBSO), including interaction tests, were estimated using multivariable Cox models.

**Results:**

Among *BRCA1pv* carriers (*n* = 403), CRRM rates were higher than in *BRCA2pv* (*n* = 317) (66% vs. 52%, *p* < 0.001) and more likely to receive timely testing, i.e., within 6 months of BC diagnosis (75% vs. 52%, *p* = 0.004). Regarding RRBSO rates, no differences were observed. CRRM was associated with significantly improved DRFS (HR = 0.63, 95% CI 0.51–0.78) and OS (HR = 0.64, 95% CI 0.51–0.82), independently of *BRCA* status and age. RRBSO was associated with improved OS only in *BRCApv* carriers, specifically, those aged ≥ 50 years (HR = 0.44, 95% CI 0.26–0.75). *BRCApv* (irrespective of affected gene) was associated with worse DRFS (HR = 1.31, 95% CI 1.06–1.63); however, this was only evident after 2 years of follow-up (HR = 1.53, 95% CI 1.22–1.93). *BRCApv* was not significantly associated with worse OS (HR = 1.25, 95%CI 0.98–1.58).

**Conclusion:**

Timely germline testing at BC diagnosis might increase CRRM rates in *BRCApv* carriers, thereby improving survival.

**Supplementary Information:**

The online version contains supplementary material available at 10.1007/s10549-025-07726-2.

## Introduction

Germline pathogenic and likely pathogenic variants in the *BRCA1* and *BRCA2* genes (*BRCApv*) account for 3–5% of breast cancer (BC) incidence and are linked to hereditary breast and ovarian cancer syndrome [1]. Female *BRCApv* carriers have a cumulative risk of BC up to 70% and ovarian cancer (OC) up to 44% [2, 3]. *BRCA1pv* carriers with stage I–III (early) unilateral BC have an increased cumulative risk of contralateral BC (CBC) of 40% compared with 17% for *BRCA2pv* carriers [2, 3]. Healthy *BRCApv* carriers are offered risk-reducing care, i.e., intensified cancer surveillance, family genetic counseling, and cancer risk-reducing surgery (RRS) [4, 5]. RRS include bilateral mastectomy, risk-reducing bilateral salpingo-oophorectomy (RRBSO), or both [6]. Knowledge of germline *BRCA* status impacts the choice of surgical treatment, e.g., patients might opt for a mastectomy instead of breast-conserving surgery combined with a contralateral risk-reducing mastectomy (CRRM) [7, 8]. CRRM is proven to reduce the risk of CBC by at least 90%, with the evidence suggesting decreased BC-specific mortality and improved overall survival (OS), albeit with some selection bias, such as “healthier people choosing or being recommended for CRRM” [9, 10]. On the contrary, *BRCA* wild-type (*BRCAwt*) carriers might opt out of a contralateral mastectomy—a procedure associated with a negative psychological impact and less clear evidence of a risk-reductive effect in this patient group [7, 11–13].

Increasing evidence advocates mainstreaming germline *BRCA* testing (hereinafter referred to as *BRCA* testing) for women with early BC to promote a risk-reducing approach [7, 14–16]. The most recent American Society of Clinical Oncology guidelines recommend *BRCA* testing in all patients with BC before the age of 65 and for selected patients over 65 [17]. However, both early and recent studies have questioned the survival benefit of CRRM for *BRCApv* carriers compared to patients with *BRCAwt* [18–20]. Furthermore, the evidence of worse survival in patients with *BRCApv*-associated BC is also conflicting, as is the evidence regarding the differing outcomes of *BRCA1pv* and *BRCA2pv* [9, 20–23].

In this nationwide and population-based retrospective study, we aimed to describe how the *BRCA* test timing and test results influenced the uptake of RRSs among females diagnosed with early BC in Denmark from 2000 to 2017. We hypothesized that timely identification of *BRCApv* (within 6 months of BC diagnosis) would lead to a higher uptake of RRSs and improved survival [24]. We compared survival between *BRCApv* and *BRCAwt* carriers using Kaplan–Meier methods and multivariable Cox models, addressing previous conflicting and potentially biased results [19–21, 23, 25–27]. By interaction tests, we examined whether *BRCA1pv* and *BRCA2pv* had different impacts on survival due to their distinct clinicopathological characteristics [1]. Finally, we explored the effects of RRSs according to *BRCA* status (*BRCApv* vs*. BRCAwt* and *BRCA1* vs. *BRCA2),* considering different BC recurrence risks and, according to age at diagnosis, addressing questionable risk-reducing effects in older (> 50 years) patients [2, 25, 28–30].

## Patients and methods

### Study setting and design

**Fig. 1:**
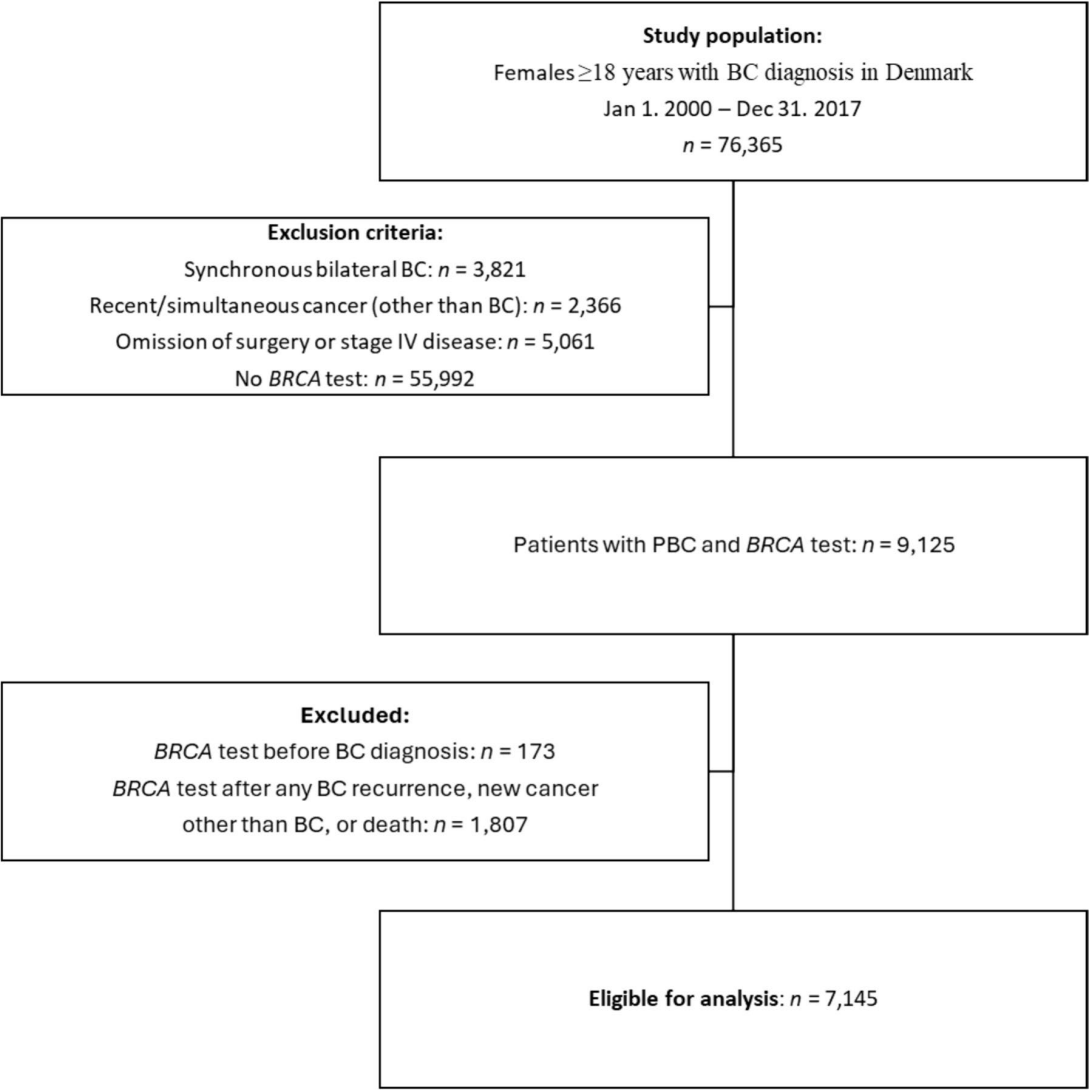
Trial profile. BC breast cancer, PBC primary breast cancer

From the clinical Danish Breast Cancer Cooperative Group (DBCG) database, we identified females (sex assigned at birth) ≥ 18 years of age with pathology-verified invasive BC between January 1, 2000, and December 31, 2017 [31]. Exclusion criteria were no registered *BRCA* test in the DBCG *BRCA* repository, synchronous bilateral BC, stage 4 disease at or within 90 days after invasive BC, omission of cancer surgery, and recent (up to 9.5 years before BC) or simultaneous (within 90 days after BC) other invasive cancer. Patients with *BRCA* tests before BC, *n* = 173, or after the first event of loco-regional recurrence, CBC, other invasive cancer, or post-mortem, *n* = 1807, were excluded (Fig. 1).

### Data sources

The clinical DBCG database provided data on primary diagnosis, immunohistochemistry, treatment, date and localization of recurrence, and follow-up time. Events of distant BC recurrence after previous loco-regional recurrence, CBC, or other malignancies were retrieved from records in the Danish Pathology Data Bank [32]. Data on definitive surgery, RRSs, and Charlson Comorbidity Index (CCI) were retrieved from the National Patient Register [33]. Missing data on baseline tumor characteristics and staging were retrieved from the Danish Pathology Data Bank.

### *BRCA* tests

*BRCA* testing was performed in five laboratories across Denmark, as previously described [34]. *BRCA* variants were classified according to the American College of Medical Genetics criteria and the Evidence-Based Network for the Interpretation of Germline Mutant Alleles [35, 36]. Patients with germline pathogenic or likely pathogenic *BRCA* variants were clinically treated equally and comprised the *BRCApv* group. Patients with *BRCA* variants of uncertain significance, likely benign or benign variants, or any variants in other screened genes included in a multigene test panel comprised the group of *BRCAwt*.

### Outcomes

The primary endpoint was distant recurrence-free survival (DRFS), defined as the time from the date of BC diagnosis to distant BC recurrence or death attributable to any cause, and complies with Standardized Definitions for Efficacy End Points version 2.0 [37]. The secondary endpoint was OS, defined as the time from BC to death of any cause. Alive and event-free patients were censored as of November 1, 2022, following a thorough update of events from multiple registries and manual revisions of electronic health records.

### Statistical analysis

All statistical analyses were performed in R for Windows, version 4.3.3. Baseline patient and tumor characteristics and treatment were summarized with counts and percentages. Categorical variables were formally tested using Chi-squared or Fisher’s exact test. Missing values were treated as separate categories under the assumption of not missing at random. Age was formally tested using the Mann–Whitney Test.

Absolute DRFS and OS estimates were analyzed using the Kaplan–Meier method, and potential median follow-up was estimated with reverse Kaplan–Meier. Left truncation at the date of the *BRCA* test result was applied to all tested patients to avoid delayed entry selection bias. CRRM and RRBSO were analyzed as time-dependent covariates to address immortal time bias. Survival curves according to type and combination of RRS were plotted using the Simon and Makuch method, assuming that RRS uptake was independent of the patient’s prognosis. We assessed the effect of *BRCApv* status and RRSs on DRFS and OS in multivariable Cox proportional hazard models adjusted for variables related to BC prognosis. Proportional hazards were assessed by a goodness-of-fit model and a formal test for independence between scaled Schoenfeld residuals and time. Time-varying effects were assessed to fulfill the proportional hazards assumption. Online Resource 1 describes the models in detail. Estimates for all variables included in the Cox models are presented in Online Resource 2 (Table 5: DRFS model) and Online Resource 3 (Table 6: OS model).

The Wald test was applied for CRRM and RRBSO differential effects in separate models, according to *BRCA* status and age (< 50 and ≥ 50 years of age at BC diagnosis). The cut-off at 50 years was chosen based on the current age criterion for *BRCA* testing in Denmark and previous research [38, 39]. *P*-values for overall effects were calculated using Likelihood-ratio tests. All *p*-values were two-sided with a significance level of 5%.

## Results

### Study cohort

Within the clinical DBCG database, we identified 7145 eligible females who, from 2000 through 2021, underwent *BRCA* testing after surgery for primary BC and before any BC recurrence, new invasive cancer, or death (Fig. 1). One patient was identified as a double *BRCA1pv* and *BRCA2pv* carrier (classified as a *BRCA1pv*), while an additional 402 had a *BRCA1pv* and 317 a *BRCA2pv* (Table 1). The 720 (10%) patients with a *BRCApv* were younger at the time of diagnosis (median age 43 vs. 47), with mainly ductal histological subtype and estrogen receptor (ER)-negative and human epidermal growth factor receptor 2 (HER2)-negative phenotype. The tumors were characterized by higher malignancy grade and larger tumor size as compared to *BRCA*wt (all *p* < 0.001). Patients with *BRCApv* more frequently had a mastectomy, more received (neo)adjuvant chemotherapy, and fewer received adjuvant endocrine therapy (ET, all *p* < 0.001). The differences between *BRCA1pv* and *BRCA2pv* patients are also presented in Table 1.

**Table 1:** Demographical, tumor, and treatment characteristics for 7145 patients with germline *BRCA* testing after PBC

	*BRCApv*				*p*-Value	*BRCA status*				*p*-Value
	*BRCA1pv*^a^		*BRCA2pv*			*BRCApv*		*BRCAwt*		
	*N*	(%)	*N*	(%)		*N*	(%)	*N*	(%)	
	403	(56)	317	(44)		720	(10)	6425	(90)	
Year of PBC diagnosis					0.18					< 0.001
2000 to 2005	84	(21)	72	(23)		156	(22)	933	(15)	
2006 to 2011	138	(34)	124	(39)		262	(36)	2124	(34)	
2012 to 2017	181	(45)	121	(38)		302	(42)	3368	(57)	
Age at PBC diagnosis (years)					< 0.001					< 0.001
18–39	174	(43)	93	(29)		267	(37)	1502	(23)	
40–59	203	(50)	175	(55)		378	(53)	3855	(60)	
≥ 60	26	(6)	49	(15)		75	(10)	1068	(17)	
Histologic type					< 0.001					< 0.001
Ductal	349	(87)	284	(90)		633	(88)	5344	(83)	
Lobular	2	(< 1)	18	(6)		20	(3)	538	(8)	
Other	50	(12)	14	(4)		64	(9)	521	(8)	
Unknown	2	(< 1)	1	(< 1)		3	(< 1)	22	(< 1)	
ER/HER2					< 0.001					< 0.001
ER–/HER2–	245	(61)	61	(19)		306	(43)	959	(15)	
ER–/HER2+	19	(5)	6	(2)		25	(3)	376	(6)	
ER+/HER2–	94	(23)	194	(61)		288	(40)	3782	(59)	
ER+/HER2+	21	(5)	27	(9)		48	(7)	887	(14)	
Unknown	24	(6)	29	(9)		53	(7)	421	(7)	
Malignancy grade					< 0.001					< 0.001
Grade I	7	(2)	27	(9)		34	(5)	1270	(20)	
Grade II	76	(19)	132	(42)		208	(29)	2544	(40)	
Grade III	258	(64)	133	(42)		391	(54)	1837	(29)	
Not graded	62	(15)	25	(8)		87	(12)	774	(12)	
Nodal status^b^					< 0.001					0.76
Node-negative	252	(63)	139	(44)		391	(54)	3608	(56)	
1–3 positive LN	96	(24)	114	(36)		210	(29)	1840	(29)	
≥ 4 positive LN	40	(10)	40	(13)		80	(11)	669	(10)	
FNA-positive	13	(3)	21	(7)		34	(5)	276	(4)	
Unknown	2	(< 1)	3	(1)		5	(1)	32	(< 1)	
Tumor size^c^					< 0.001					< 0.001
0–10 mm	32	(8)	52	(16)		84	(12)	1200	(19)	
11–20 mm	181	(45)	105	(33)		286	(40)	2694	(42)	
21–50 mm	170	(42)	142	(45)		312	(43)	2210	(34)	
≥ 50 mm	20	(5)	17	(5)		37	(5)	282	(4)	
Unknown	0	(0)	1	(< 1)		1	(< 1)	39	(1)	
Definitive surgery^d^					< 0.001					< 0.001
Mastectomy	137	(34)	69	(22)		206	(29)	1273	(20)	
Mastectomy with RT	85	(21)	108	(34)		193	(27)	1619	(25)	
Breast-conserving with RT	181	(45)	140	(44)		321	(45)	3533	(55)	
(Neo)adjuvant chemotherapy^e^					< 0.001					< 0.001
Any administered	362	(90)	246	(78)		608	(84)	4689	(73)	
No	41	(10)	71	(22)		112	(16)	1736	(27)	
Endocrine therapy					< 0.001					< 0.001
Any administered	115	(29)	214	(68)		329	(46)	4306	(67)	
No	288	(71)	103	(32)		391	(54)	2119	(33)	
Comorbidity^f^					0.08					0.30
CCI 0	357	(89)	285	(90)		642	(89)	5670	(88)	
CCI 1	34	(8)	16	(5)		50	(7)	542	(8)	
CCI ≥ 2	12	(3)	16	(5)		28	(4)	213	(3)	
Follow-up, median years (range)	11.6	(8.4–16.2)	12.4	(8.9–16.3)		12.0	(8.5–16.3)	10.6	(7.5–14.5)	
Death	60	(15)	60	(19)		120	(17)	721	(11)	
Ovarian cancer	7	(2)	1	(< 1)		8	(1)	7	(< 1)	
Age, median years (range)	57.2	(55.0–69.5)	56.5	(56.5–56.5)		56.9	(55.3–67.8)	61.6	(60.3–68.2)	

*CCI* Charlson comorbidity index, *DBCG* Danish breast cancer cooperative group, *ER–* estrogen receptor-negative, *HER2–/* + human epidermal growth factor receptor-negative/positive, *ICD-8/-10* international classification of diseases, revision 8/10, *LN* lymph nodes, *PBC* primary breast cancer, *RT* radiotherapy

^a^One patient is double-carrier, with both *BRCA1pv* and *BRCA2pv*, but is classified as *BRCA1pv*

^b^The clinical node status was applied (radiologically determined with ultrasound or magnetic resonance imaging) or fine-needle aspirate from axillary nodes for patients who had received neoadjuvant chemotherapy. Otherwise, the pathological nodal status was applied. If axillary dissection was performed and no LNs were found, the nodal status was determined as “unknown”

^c^For patients who had received neoadjuvant chemotherapy, the clinical tumor size (radiologically determined by ultrasound or magnetic resonance imaging) was applied. Pathological tumor size was applied for patients with upfront surgery

^d^Intention-to-treat RT according to DBCG guidelines

^e^Includes administered anti-HER2 treatment

^f^CCI was calculated using ICD-8 and ICD-10 codes for 19 chronic diseases retrieved from hospital records in the 10 years before the BC diagnosis (see Online resource 1 for further assumptions)

### *BRCA* test timing and risk-reducing surgery

Among 7145 patients, 2169 (30%) received their *BRCA* test results within 6 months of BC diagnosis, thus representing timely testing (Table 2). Among those with timely testing, 154 (7%) had a *BRCA1pv,* while 88 (4%) had a *BRCA2pv*. RRS, either CRRM, RRBSO, or both, was conducted in 1677 (26%) of patients with *BRCAwt* compared to 369 (92%) of patients with *BRCA1pv* and 283 (89%) with *BRCA2pv*. Ninety-two of 181 patients (51%) with *BRCA1pv* and 53 of 140 (38%) with *BRCA2pv* opted for a subsequent bilateral mastectomy after initial breast-conserving surgery (data not shown). The uptake of RRBSO was similar (82%) in *BRCA1pv* and *BRCA2pv* carriers and there was no difference depending on test timing (*p* > 0.99). In contrast, the uptake of CRRM was higher in *BRCA1pv* compared to *BRCA2pv* (66% vs. 52%, *p* < 0.001) and higher if timely tested (75% vs. 52%, *p* = 0.004).

**Table 2:** Proportions of patients with risk-reducing surgery according to *BRCA* test timing and the test result

						Risk-reducing surgery								
	N (%)	Median age at PBC diagnosis (IQR)	Median time from PBC to genetic testing, weeks (IQR)	Median time from PBC to CRRM, weeks (IQR)	Median time from PBC to RRBSO, weeks (IQR)	CRRM and RRBSO		CRRM only		RRBSO only		None		*p*-Value^a^
						*N*	(%)	*N*	(%)	*N*	(%)	*N*	(%)	
*BRCA* test <6 months^b^	2169 (30%)													0.009
*BRCA1pv*	154 (7%)	39 (33–48)	14 (9–18)	34 (29–80)	45 (37–65)	105	(68)	21	(14)	18	(12)	10	(6)	
*BRCA2pv*	88 (4%)	44 (37–53)	15 (10–20)	43 (31–88)	40 (30–55)	45	(51)	10	(11)	24	(27)	9	(10)	
*BRCA*wt	1927 (89%)	44 (38–52)	15 (10–20)	52 (29–106)	68 (41–147)	75	(4)	228	(12)	261	(14)	1363	(71)	
*BRCA* test >6 months^c^	4976 (70%)													0.40
*BRCA1pv*	249 (5%)	42 (36–49)	65 (38–284)	101 (67–182)	88 (56–239)	121	(49)	18	(7)	86	(35)	24	(10)	
*BRCA2pv*	229 (5%)	46 (39–56)	79 (41–249)	127 (76–319)	96 (58–258)	96	(42)	14	(6)	94	(41)	25	(11)	
*BRCA*wt	4498 (90%)	48 (41–56)	90 (43–333)	95 (49–159)	120 (56–264)	129	(3)	243	(5)	741	(16)	3385	(75)	

*BRCAwt* wild-type *BRCA1* and *BRCA2* genes, *BRCApv* pathogenic variants in the *BRCA1* and *BRCA2* genes, *CRRM* contralateral risk-reducing mastectomy, *PBC* primary breast cancer, *RRBSO* risk-reducing bilateral salpingo-oophorectomy

^a^Chi-squared test for distributions of risk-reducing surgery among *BRCA1pv* vs. *BRCA2pv*

^b^Mann–Whitney U test for differences in age at PBC: *BRCA1pv* vs. *BRCAwt p* < 0.0001, *BRCA2pv* vs. *BRCAwt p* = 0.86

^c^Mann–Whitney U test for differences in age at PBC: *BRCA1pv* vs. *BRCAwt p* < 0.0001, *BRCA2pv* vs. *BRCAwt p* = 0.03

### Survival analysis

**Fig. 2:**
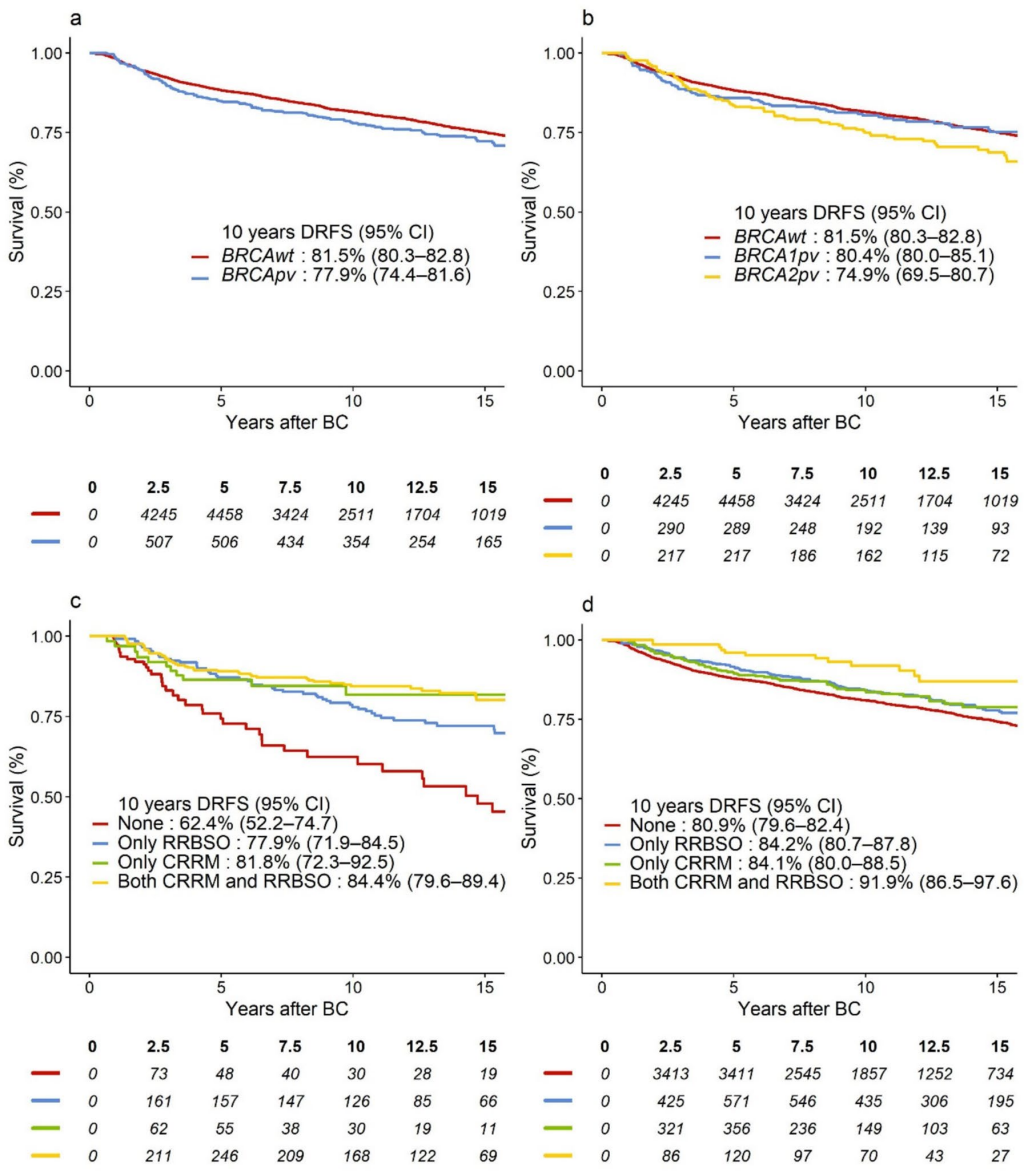
Survival curves representing DRFS in **a** *BRCApv* vs. *BRCAwt* carriers; **b** *BRCA1pv* vs. *BRCA2pv* vs. *BRCAwt* carriers; DRFS-curves according to type and combination of risk-reducing surgery for **c** *BRCApv* carriers, **d** *BRCAwt* carriers. *BRCAwt* wild-type *BRCA1* and *BRCA2* genes, *BRCApv* pathogenic variants in the *BRCA1* and *BRCA2* genes, BC breast cancer, CI confidence interval, CRRM contralateral risk-reducing mastectomy, DRFS distant recurrence-free survival, RRBSO risk-reducing bilateral salpingo-oophorectomy

**Fig. 3:**
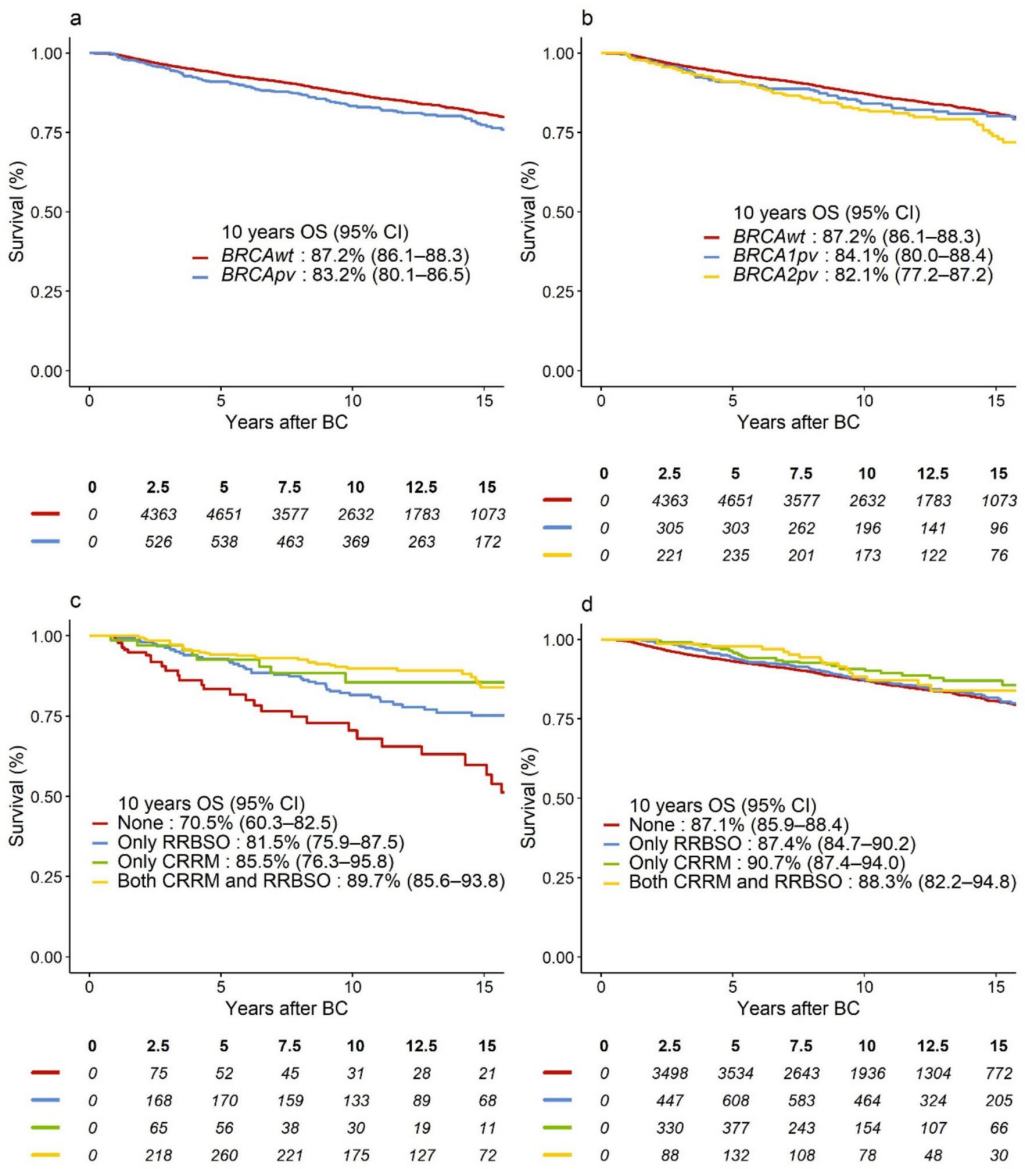
Survival curves representing OS in **a** *BRCApv* vs. *BRCAwt* carriers; **b** *BRCA1pv* vs. *BRCA2pv* vs. *BRCAwt* carriers; OS-curves according to type and combination of risk-reducing surgery for **c** *BRCApv carriers*, **d** *BRCAwt* carriers. *BRCAwt* wild-type *BRCA1* and *BRCA2* genes, *BRCApv* pathogenic variants in the *BRCA1* and *BRCA2* genes, BC breast cancer, CI confidence interval, CRRM contralateral risk-reducing mastectomy; OS overall survival; RRBSO risk-reducing bilateral salpingo-oophorectomy

The potential median follow-up was 10.8 years. Of 1104 events in the DRFS analysis, 785 (71%) were distant BC recurrences and 319 (29%) were deaths as the first event. There were 841 events in the OS analysis. In unadjusted analysis, we observed 3.6% lower absolute 10-year DRFS and 4.0% lower 10 year OS for *BRCApv* vs. *BRCA*wt (Figs. 2a and 3a).

When adjusted for baseline characteristics and treatment, including RRS, *BRCApv* status was associated with significantly worse DRFS, hazard ratio (HR) = 1.31 (95% confidence interval [CI] 1.06–1.63), *p* = 0.01. However, this effect was mainly evident after 2 years of follow-up (HR = 1.53, 95% CI 1.22–1.93), while *BRCApv* did not significantly worsen the prognosis in years 0–2 (HR = 0.72, 95% CI 0.47–1.11), heterogeneity *p* < 0.001 (Table 3). In the adjusted analysis, *BRCApv* was associated with worse OS (HR = 1.25, 95% CI 0.99–1.59), which was similarly observed in Fig. 3a. However, this relation was not statistically significant, *p* = 0.07. Although *BRCA2pv* carriers exhibited the lowest absolute 10-year DRFS and OS (Figs. 2b and 3b, respectively), as compared to *BRCA1pv*, *BRCA2pv* was not associated with worse DRFS (heterogeneity *p* = 0.64, Table 3) or OS (heterogeneity *p* = 0.35, Table 4). Tables 5 (Online Resource 2) and Table 6 (Online Resource 3) show overall and adjusted HR estimates for all variables included in the models.

**Table 3:** Multivariable Cox model estimates for DRFS, showing adjusted HR and 95% CI for the overall effect of *BRCApv* vs. *BRCAwt* and for CRRM yes vs. no, and RRBSO yes vs. no. Time-varying effects (early vs. late and corresponding heterogeneity *p*-value) of *BRCA status* adjusting for non-proportional hazards

	Overall effect Adjusted HR (95% CI)^a^	*p*-Value	Early effect adjusted HR (95% CI)	Late effect adjusted HR (95% CI)	*p*-Value for heterogeneity
*BRCA* status^b^					
*BRCApv vs. BRCAwt*	1.31 (1.06–1.63)	0.01	0.72 (0.47–1.11)	1.53 (1.22–1.93)	< 0.001
Subgroup analysis:					
*BRCA1pv vs. BRCAwt*	1.16 (0.88–1.54)		0.71 (0.42–1.20)	1.39 (1.01–1.90)	0.64
*BRCA2pv vs. BRCAwt*	1.47 (1.13;1.91)		0.73 (0.36–1.49)	1.66 (1.26–2.18)	
CRRM					
Yes vs. No	0.63 (0.51–0.78)	< 0.001			
Interactions CRRM					
*BRCA*wt	0.72 (0.56–0.93)				0.06
*BRCApv*	0.49 (0.35–0.70)				
*BRCA1pv*	0.38 (0.23–0.62)				0.12
*BRCA2pv*	0.65 (0.40–1.04)				
*BRCAwt* < 50 years	0.66 (0.49–0.88)				0.14
*BRCAwt* ≥ 50 years	0.98 (0.63–1.55)				
*BRCApv* < 50 years	0.47 (0.32–0.69)				0.58
*BRCApv* ≥ 50 years	0.57 (0.31–1.05)				
RRBSO					
Yes vs. No	0.89 (0.75–1.06)	0.20			
Interactions RRBSO					
*BRCA*wt	0.96 (0.79–1.16)				0.12
*BRCApv*	0.70 (0.50–0.99)				
*BRCA1pv*	0.68 (0.42–1.11)				0.86
*BRCA2pv*	0.73 (0.45–1.18)				
*BRCAwt* < 50 years	0.96 (0.75–1.24)				0.94
*BRCAwt* ≥ 50 years	0.98 (0.73–1.30)				
*BRCApv* < 50 years	0.75 (0.51–1.09)				0.54
*BRCApv* ≥ 50 years	0.66 (0.41–1.04)				

*p*-Values for heterogeneity correspond to tests for differential effects of CRRM and RRBSO according to *BRCA* status and age and for differences in *BRCA1pv* and *BRCA2pv* estimates in a subgroup analysis

*BRCAwt* wild-type *BRCA1* and *BRCA2* genes, *BRCApv* pathogenic variants in the *BRCA1* and *BRCA2* genesl, *CI* confidence intervall, *CRRM* contralateral risk-reducing mastectomy, *DRFS* distant recurrence-free survival, *ER* estrogen receptor, *HER2* human epidermal growth factor receptor, *HR* hazard ratio, *RRBSO* risk-reducing bilateral salpingo-oophorectomy

^a^Variables included in the model: *BRCA* status (*BRCApv* vs. *BRCAwt*), CRRM (time-dependent), RRBSO (time-dependent), age at diagnosis, Charlson Comorbidity Index, definitive surgery method, histological subtype, malignancy grading, tumor diameter, ER/HER2 status, nodal status, (neo)adjuvant chemotherapy and adjuvant endocrine therapy

^b^Early (0–2 years) and late (> 2 years of follow-up) effects of *BRCApv*, including *BRCA1pv* and *BRCA2pv,* are presented to comply with the proportional hazard assumption

**Table 4:** Multivariable Cox model estimates for overall survival, showing adjusted HR and 95% CI for the overall effect of *BRCApv* vs. *BRCAwt* and for CRRM yes vs. no, and RRBSO yes vs. no

	Overall effect adjusted HR (95% CI)^a^	*p*-Value	*p*-Value for heterogeneity
*BRCA* status			
*BRCApv vs. BRCAwt*	1.25 (0.98–1.58)	0.07	
Subgroup analysis			
*BRCA1pv vs. BRCAwt*	1.12 (0.82–1.53)		0.29
*BRCA2pv vs. BRCAwt*	1.37 (1.02–1.84)		
CRRM			
Yes vs. No	0.64 (0.51–0.82)	< 0.001	
Interactions CRRM			
*BRCA*wt	0.76 (0.57–1.00)		0.07
*BRCApv*	0.49 (0.33–0.72)		
*BRCA1pv*	0.38 (0.23–0.65)		0.16
*BRCA2pv*	0.66 (0.39–1.13)		
*BRCAwt* < 50 years	0.68 (0.49–0.96)		0.20
*BRCAwt* ≥ 50 years	1.00 (0.61–1.64)		
*BRCApv* < 50 years	0.47 (0.31–0.72)		0.44
*BRCApv* ≥ 50 years	0.62 (0.32–1.21)		
RRBSO			
Yes vs. No	0.98 (0.82–1.18)	0.84	
Interactions RRBSO			
*BRCA*wt	1.09 (0.89–1.33)		0.03
*BRCApv*	0.67 (0.46–0.98)		
*BRCA1pv*	0.76 (0.44–1.31)		0.51
*BRCA2pv*	0.59 (0.35–1.00)		
*BRCAwt* < 50 years	1.33 (1.03–1.72)		0.03
*BRCAwt* ≥ 50 years	0.85 (0.61–1.18)		
*BRCApv* < 50 years	0.87 (0.58–1.31)		0.01
*BRCApv* ≥ 50 years	0.44 (0.26–0.75)		

*p*-Values for heterogeneity correspond to tests for differential effects of CRRM and RRBSO according to *BRCA* status and age and for differences in *BRCA1pv* and *BRCA2pv* estimates in a subgroup analysis

*BRCAwt* wild-type *BRCA1* and *BRCA2* genes, *BRCApv* pathogenic variants in the *BRCA1* and *BRCA2* genes, *CI* confidence interval, *CRRM* contralateral risk-reducing mastectomy, *ER* estrogen receptor, *HER2* human epidermal growth factor receptor, *HR* hazard ratio, *RRBSO* risk-reducing bilateral salpingo-oophorectomy

^a^Variables included in the model: *BRCA* status (*BRCApv* vs. *BRCAwt*), CRRM (time-dependent), RRBSO (time-dependent), age at diagnosis, Charlson Comorbidity Index, definitive surgery method, histological subtype, malignancy grading, tumor diameter, ER/HER2 status, nodal status, (neo)adjuvant chemotherapy and adjuvant endocrine therapy

In the unadjusted analysis*, BRCApv* carriers who received any RRS showed improved 10 year survival (Figs. 2c and 3c) compared to those who opted out of RRS, while the risk-reducing effect of CRRM and RRBSO seemed less prominent for *BRCAwt* carriers (Figs. 2d and 3d). The most significant improvement in survival was observed in *BRCApv* carriers who opted for CRRM (alone or with RRBSO). The rate of CBC was 2% among those who opted for CRRM (26 of 1105) vs. 4% in the non-CRRM group (217 of 6040). In multivariable analysis, CRRM was associated with significantly better DRFS (adjusted HR = 0.63, 95% CI 0.51–0.78); *p* < 0.001) and better OS (adjusted HR = 0.64, 95% CI 0.51–0.81), *p* < 0.001. As shown in Tables 3 and 4, several interactions were explored. Interaction tests could not reject the null hypothesis of no difference in effects of CRRM in *BRCApv* carriers vs. *BRCAwt*, or in *BRCA1* vs. *BRCA2* (all heterogeneity *p* > 0.05). RRBSO was not significantly associated with DRFS or OS (*p* = 0.20 and *p* = 0.82, respectively) in the multivariable analyses. However, interaction tests revealed that RRBSO was associated with OS benefit for *BRCApv* (HR = 0.67, 95% CI 0.46–0.98), but not for *BRCAwt* (heterogeneity *p* = 0.03), and without differences according to the affected gene (heterogeneity *p* = 0.51, Table 4).

### Patients ≥ 50 years of age at diagnosis

In the multivariable models, we tested for different effects of CRRM and RRBSO according to age at diagnosis and *BRCA* status. In *BRCApv* patients, CRRM was associated with improved DRFS regardless of age, showing HR = 0.47, 95% CI 0.32–0.69 and HR = 0.57, 95% CI 0.31–1.05, for those < 50 and ≥ 50 years of age at BC diagnosis, respectively, heterogeneity *p* = 0.58. Similarly, interaction tests showed no difference in CRRM effects for *BRCAwt* according to age, heterogeneity *p* = 0.14. In the OS model, tests for interaction between CRRM and age returned similar results (Table 4). RRBSO was associated with significantly improved OS for *BRCApv* carriers ≥ 50 years at diagnosis with HR = 0.44, 95% CI 0.26–0.75. Although RRBSO also decreased hazards for those < 50 at diagnosis, the effect was not significant, showing HR = 0.87, 95% CI 0.58–1.31, heterogeneity *p* = 0.01.

## Discussion

In this large national cohort of 7145 females with primary BC, we observed an association between germline *BRCA* test timing and the uptake of RRS. Few *BRCApv* carriers (8–11%) completely opted out of RRSs, and around 80% opted for RRBSO irrespective of test timing and the affected gene. The uptake of CRRM was correspondingly high among timely tested *BRCA1pv* but was for *BRCA2pv* somewhat lower, and late testing reduced the uptake in both groups. The median time from PBC to genetic testing was approximately 15 weeks for timely tested patients, and the median time to CRRM was 34–43 weeks for timely tested *BRCApv* carriers. Patients with *BRCApv* (10%) had significantly higher hazards for events of distant BC relapse or death (DRFS) only after 2 years of follow-up (HR = 1.53, 95% CI 1.22–1.93). *BRCApv* carriers tended to have worse prognostic characteristics, including larger tumors, higher malignancy grading, and ER-negative/HER2-negative subtype. The multivariable analysis did not find a significant association with OS (HR = 1.25, 95% CI 0.98–1.58, *p* = 0.07), suggesting that the presence of *BRCApv* may not independently worsen survival outcomes. Instead, the prognosis highly depends on other factors included in the models, such as age, CCI, tumor characteristics and treatment. Patients with *BRCA*-associated BC were more heavily treated, i.e., received bilateral mastectomy and (neo)adjuvant chemotherapy more often, compared with *BRCAwt*, which might have improved the prognosis. Compared with *BRCAwt*, *BRCA2pv* was associated with worse survival outcomes in multivariable models but was not significantly different from *BRCA1pv.* Our results align with OS estimates from a meta-analysis by Liu et al. [21]. They found that although *BRCA1pv* was associated with worse OS compared to *BRCA1wt* (HR = 1.20, 95% CI 1.08–1.33; *p* = 0.0008) with worse outcomes in ≤ 5 years follow-up, and *BRCA2pv* with worse OS after 5 years of follow-up (HR = 1.39, 95% CI 1.22–1.58), combined *BRCA1/2pv* was not significantly associated with worse OS (HR = 0.98, *p* = 0.84) [21].

After being diagnosed with primary BC, women and their physicians’ primary concerns are the risks of disease recurrence and death [40]. Only a few tools have been developed and validated for predicting CBC risk, but none are currently implemented in national guidelines [41–43]. An increasing amount of BC patients opt for RRSs, with proven risk-reducing effects in *BRCApv* carriers and questionable effects in *BRCA*wt patients [6, 9, 10, 17, 19, 20, 39, 44–47]. The evidence of *BRCA* test timing on survival is poorly described, but studies show a coherence between *BRCA* testing at the time of BC diagnosis and surgical treatment decision, similar to our findings [8, 48, 49]. Only a few patients (*n* = 173) knew their germline *BRCA* status before PBC, of whom 44 (25%) opted out of bilateral mastectomy. By contrast, 225 patients (3%) opted for CRRM before *BRCA* testing, of whom 28 (12%) had *BRCA1pv*, 17 (8%) had *BRCA2pv*, and 180 (80%) were *BRCAwt*. This indicates that most Danish patients were genetically counselled prior to CRRM. In a previous cohort study, we have demonstrated that patients under 40 years of age at PBC and those with ER-negative/HER2-negative BC received *BRCA* testing more often, while the majority of patients still missed testing [34]. The survival benefit of CRRM that we observed among *BRCApv* patients was similar to previous findings and did not seem restricted to *BRCA1* or young age (< 50) at diagnosis [6, 9, 45, 50, 51]. However, the OS benefit associated with RRBSO among *BRCApv* patients who were 50 years and older at primary diagnosis reflects mainly the prevention of secondary OC and less the endocrine suppression effect [51, 52]. Our results are of great importance for clinicians when discussing risk-reducing procedures with patients 50 years and older at BC diagnosis [8].

A Cochrane review found no evidence of survival benefit after CRRM among *BRCA*wt patients [5]. In contrast, our results suggest that CRRM might also reduce hazards for events of distant BC recurrence and death among these high-risk patients. Although the effects of RRSs were less pronounced in *BRCA*wt patients, we found only significantly lower effects of RRBSO in the OS model since these patients might have a lower risk of OC. However, given the low uptake of CRRM (11%) among *BRCAwt* patients, the results for this group should be interpreted with caution. Furthermore, we miss information on patients who chose to undergo RRSs due to pathogenic variants in other high-risk genes such as *TP53, STK11, PTEN, PALB2,* and *CDH1* or after pedigree-based BC risk assessment. The *BRCA*wt population in our study had a higher risk for recurrence than sporadic BC since they were referred to *BRCA* testing, which might explain the observed benefit from CRRM. During the study period, moderate cancer risk genes such as *ATM, CHEK2*, and *BARD1* were not analyzed in Denmark [38, 53]. The uptake of CRRM in high-risk *BRCA*wt young females with BC in Denmark is high and comparable to this in other countries [7, 38, 54].

Sixty-one percent of *BRCA1pv* carriers in our cohort were ER- and HER2-negative, which is an indication for (neo-)adjuvant chemotherapy [55]. Most (90%) of *BRCA1pv*, 78% of *BRCA2pv*, and 73% of *BRCA*wt patients received chemotherapy (CT). CT was associated with significantly reduced hazards for both DRFS and OS (Online Resources 2 and 3). The reduced administration rate of CT might partially explain the poorer prognosis of *BRCA2*-associated BC, which was most often ER-positive (70%). Even though most of the *BRCA2pv* carriers with ER-positive BC received adjuvant ET, non-adherence to ET is evident among patients with this BC subtype, with an increased risk for relapse and death [56]. Furthermore, ER-positive status might have counterintuitive results in certain patient subgroups with mixed results reported [57–59]. Newer therapies for ER-positive BC, such as inhibitors of cyclin-dependent kinase 4 and 6 and intensified focus on ET compliance, might benefit this subgroup [60]. Four out of five *BRCApv* carriers in our cohort had HER2-negative disease and had received CT and are potential candidates for adjuvant treatment with poly-ADP-ribose-polymerase inhibitors, such as olaparib [61]. However, olaparib is not currently approved in Denmark for this indication, leaving most of these patients without a targeted systemic treatment.

### Strengths and limitations

The strength of our study lies in the evaluation of long-term outcomes for 720 *BRCApv* patients compared to 6425 *BRCAwt* patients who underwent *BRCA* testing after BC diagnosis with a median follow-up of 10.8 years. The integration of several national registries allowed us to analyze the timing of *BRCA* testing and the effects of test results on RRS decisions and survival. Missing data was primarily on HER2 prior to 2006 (Supplementary Methods, Online Resource 1). We assessed the effects of *BRCApv* and RRSs on time to distant BC recurrence or death (DRFS) and OS in multivariable Cox analysis, adjusting for known prognostic factors to minimize bias, as previously recommended [10, 62]. We also tested for differential effects of RRSs depending on the *BRCA* status and age at diagnosis, as hypothesized. In contrast, previous studies failed to analyze the effect of *BRCA* status using multivariable Cox regression models [19, 63]. Our primary outcome is clinically relevant, since distant BC recurrence is associated with the worst prognosis [21]. Furthermore, DRFS does not account for events of local or contralateral BC recurrence, as assessing the risk of CBC in the case of CRRM is biased by missing the organ at risk, as discussed in a large Surveillance, Epidemiology, and End Results study [64]. While a meta-analysis has examined the association between *BRCA* status and distant metastasis-free survival, an outcome equivalent to our primary outcome, the studies included in the distant metastasis-free survival analysis reported distant disease-free survival, which also accounts for the time to other non-breast, mainly ovarian cancers [21]. One limitation of our study is that, despite extensive data integration, not all participants had systematic follow-up at hospital departments reporting to DBCG. This could potentially bias the prolonged follow-up time for patients who were alive and event-free. Another limitation is the inclusion of patients with pathogenic variants in other high-risk genes as *BRCAwt*, which might attenuate the results for this patient group. The median OS was not reached during the follow-up, which might account for the non-significant survival differences between *BRCApv* and *BRCAwt* [65]. Lastly, patients in our cohort were selected for germline *BRCA* testing, and this selection may, at least in part, explain the benefit of CRRM in *BRCAwt* as well as *BRCApv* carriers.

## Conclusion

In conclusion, *BRCApv* status was associated with significantly worse DRFS (HR = 1.31, 95% CI 1.06–1.63, *p* = 0.01) but not with OS (HR = 1.25, 95% CI 0.98–1.58, *p* = 0.07). CRRM was associated with survival benefits mainly among *BRCApv* carriers and regardless of the age at diagnosis, while RRBSO was primarily effective in *BRCApv* carriers over 50 years of age at BC diagnosis, presumably reflecting a reduction in the risk of OC. *BRCA2pv* status was linked to delayed testing and lower CRRM and adjuvant chemotherapy rates; however, in the adjusted analysis, *BRCA2pv* outcomes were not significantly worse compared to *BRCA1pv*. Our results highlight the importance of prompt *BRCA* testing for women with BC, irrespective of age and hormone receptor status, to identify *BRCApv* carriers and enable timely RRS and targeted systemic treatment [61, 66].

## Supplementary Information

Below is the link to the electronic supplementary material.

Supplementary file1 (DOCX 17 KB)

Supplementary file2 (DOCX 30 KB)

## Data Availability

The findings of this study are supported by data which can be provided upon request and approval from the Danish Breast Cancer Group. Data may not be publicly available due to institutional restrictions and by the Danish Health Law.

## Abbreviations

BC: Breast cancer
*BRCApv*: Pathogenic variants in the *BRCA1* and *BRCA2* genes
*BRCAwt*: Wild-type *BRCA1* and *BRCA2* genes
CBC: Contralateral breast cancer
CCI: Charlson comorbidity index
CI: Confidence interval
CRRM: Contralateral risk-reducing mastectomy
CT: Chemotherapy
DBCG: Danish breast cancer cooperative group
DMFS: Distant metastasis-free survival
DRFS: Distant recurrence-free survival
ER: Estrogen receptor
ET: Endocrine therapy
HER2: Human epidermal growth factor receptor 2
HR: Hazard ratio
LN: Lymph nodes
MRI: Magnetic resonance imaging
OC: Ovarian cancer
OS: Overall survival
PBC: Primary breast cancer
RRBSO: Risk-reducing bilateral salpingo-oophorectomy
RRS: Risk-reducing surgery
RT: Radiotherapy
