# Supplementary material for: Timely germline BRCA testing after invasive breast cancer promotes contralateral risk-reducing mastectomy and improves survival: an observational retrospective study
Source: Breast Cancer Res Treat. 2025 May 23;212(2):309–23. doi: 10.1007/s10549-025-07726-2 (PMC12134039; doi:10.1007/s10549-025-07726-2)
Supplement: Supplementary file 1 — Supplementary file1 (DOCX 17 KB) [file 10549_2025_7726_MOESM1_ESM.docx]

**Online Resource 1**

**Supplementary methods**

Variables with a known impact on breast cancer survival were evaluated in univariate Cox models, and missing values were parameterized accordingly. Tumors with other/unknown histological subtypes were assigned malignancy grade 1, while ungraded ductal and lobular carcinomas were assigned grade 2 [1]. Unknown tumor size was assigned to ≤20 mm. Unknown nodal status was assigned to node-negative and fine-needle aspirate-positive axillary status, and ≥ four positive lymph nodes were analyzed as one group. Unknown receptor subtype includes unknown estrogen receptor (*n* = 10) and unknown human epidermal growth factor receptor 2 (*n* = 464).

Charlson comorbidity index (CCI) was calculated using ICD-8 and ICD-10 codes for 19 chronic diseases retrieved from the National Patient Registry (NPR) from both in and outpatient hospital records in the 10 years before the BC diagnosis. Patients with chronic diseases treated by their general practitioner without hospitalization are not registered in NPR. Patients for whom there were no previous hospital records for the relevant diseases were assigned to CCI 0.

A goodness-of-fit model and a formal test for independence between scaled Schoenfeld residuals and time for the proportional hazards’ assumption identified variables with time-varying coefficients. The overall effects are not adjusted for non-proportionality. Follow-up was split into intervals at cut-points 2, 5, and 10 years to comply with the proportional hazards assumption of the Cox regression model [2]. Tests of time-varying coefficient heterogeneity were conducted in univariate and multivariable models. Variables exhibiting more than one time-varying effect were assessed at several time-points. Overall tests for proportional hazards in the final multivariable Cox models, including time-varying coefficients, all returned *p* > 0.10.

**References**

1. Rakha EA, Reis-Filho JS, Baehner F, Dabbs DJ, Decker T, Eusebi V, et al. Breast cancer prognostic classification in the molecular era: the role of histological grade. *Breast Cancer Research*. 2010;12(4):207. DOI: <https://doi.org/10.1186/bcr2607>

Therneau T, Crowson C, Atkinson E. Using time dependent covariates and time dependent coefficients in the cox model. 2014. Available from: <https://cran.r-project.org/web/packages/survival/vignettes/timedep.pdf>. Accessed 13 January, 2025.
