# Supplementary material for: Timely germline BRCA testing after invasive breast cancer promotes contralateral risk-reducing mastectomy and improves survival: an observational retrospective study
Source: Breast Cancer Res Treat. 2025 May 23;212(2):309–23. doi: 10.1007/s10549-025-07726-2 (PMC12134039; doi:10.1007/s10549-025-07726-2)
Supplement: Supplementary file 2 — Supplementary file2 (DOCX 30 KB) [file 10549_2025_7726_MOESM2_ESM.docx]

**Online resource 2**

**Table 5** Multivariable Cox proportional hazards model for time to distant BC recurrence or death with adjusted HR and 95% CI for overall and time-varying (initial and late) effects

|  |  | **Overall effect**  Adjusted HR, (95% CI) | *p*-value for overall effect | **Initial effect^a^**  Adjusted HR, (95% CI) | **Late effect**  Adjusted HR,(95% CI) | Heterogeneity *p*-value |
| --- | --- | --- | --- | --- | --- | --- |
| ***BRCA* status** | | Ref = *BRCA*wt | 0.01 |  |  |  |
|  | *BRCApv* | 1.31 (1.06–1.63) |  | 0.72 (0.47–1.11) | 1.53 (1.22–1.93) | 0.002 |
| **CRRM** | | Ref = No | <0.001 |  |  |  |
|  | Yes (time-dependent) | 0.63 (0.51–0.78) |  | 0.63 (0.51–0.78) |  |  |
| **RRBSO** | | Ref = No | 0.09 |  |  |  |
|  | Yes (time-dependent) | 0.86 (0.73–1.02) |  | 0.89 (0.75–1.06) |  |  |
| **Age at diagnosis (years)** | | Ref = 40–59 | <0.001 |  |  |  |
|  | 18–39 | 1.17 (1.01–1.35) |  | 1.49 (1.24–1.79) | 0.78 (0.61–0.99) | <0.001 |
|  | ≥60 | 1.54 (1.30–1.83) |  | 1.05 (0.81–1.37) | 2.17 (1.74–2.69) | <0.001 |
| **CCI** | | Ref = CCI 0 | <0.001 |  |  |  |
|  | CCI 1 | 1.22 (1.00–1.50) |  | 0.95 (0.69–1.31) | 1.57 (1.21–2.04) | 0.02 |
|  | CCI 2+ | 1.76 (1.35–2.29) |  | 1.79 (1.38–2.33) |  |  |
| **Definitive surgery** | | Ref = Mastectomy -RT | <0.001 |  |  |  |
|  | Breast-conserving | 0.61 (0.50–0.73) |  | 0.55 (0.45–0.67) | 0.80 (0.59–1.07) | 0.02 |
|  | Mastectomy +RT | 0.94 (0.75–1.18) |  | 0.93 (0.74–1.17) |  |  |
| **Histological subtype** | | Ref = Ductal | 0.73 |  |  |  |
|  | Lobular | 1.05 (0.84–1.30) |  | 1.07 (0.86–1.33) |  |  |
|  | Other or unknown | 0.91 (0.67–1.22) |  | 1.54 (0.97–2.45) | 0.77 (0.54–1.10) | 0.01 |
| **Malignancy grading** | | Ref = Grade 1 | <0.001 |  |  |  |
|  | Grade 2 | 1.29 (1.06–1.56) |  | 1.29 (1.06–1.56) |  |  |
|  | Grade 3 | 1.55 (1.25–1.93) |  | 1.84 (1.43–2.36) | 1.41 (1.08–1.83) | 0.06 |
| **Tumor diameter** | | Ref = ≤20mm | <0.001 |  |  |  |
|  | >20 mm | 1.34 (1.17–1.53) |  | 1.44 (1.24–1.68) | 0.94 (0.70–1.26) | 0.009 |
| **ER/HER2** | | Ref = ER+/HER2– | <0.001 |  |  |  |
|  | TNBC | 0.89 (0.70–1.12) |  | 1.08 (0.74–1.58) | 0.65 (0.49–0.85) | 0.02 |
|  | HER2+ | 0.65 (0.54–0.78) |  | 0.63 (0.52–0.76) |  |  |
|  | Unknown | 0.58 (0.43–0.78) |  | 0.53 (0.39–0.70) |  |  |
| **Nodal status** | | Ref = Node-negative | <0.001 |  |  |  |
|  | 1–3 positive nodes | 1.44 (1.20–1.73) |  | 1.39 (1.16–1.67) |  |  |
|  | ≥4 positive nodes/FNA-positive | 2.88 (2.36–3.51) |  | 3.82 (2.80–5.21) | 2.53 (2.05–3.14) | 0.001 |
| **(Neo)adjuvant chemotherapy** | | Ref = Not administered | <0.001 |  |  |  |
|  | Any | 0.69 (0.59–0.82) |  | 0.85 (0.70–1.03) | 0.51 (0.38–0.68) | 0.002 |
| **Adjuvant endocrine therapy** | | Ref = Not administered | <0.001 |  |  |  |
|  | Any (0–2 years) | 0.66 (0.55–0.78) |  | 0.26 (0.18–0.38) | 0.48 (0.37–0.62) | 0.006 |
|  | Any (>5 years) |  |  |  | 1.06 (0.85–1.33) | <0.001 |

^a^Variables showing time-varying effects:
At 2 years*: BRCA*pv, histological subtype other/unknown, TNBC, ≥4 positive nodes/FNA-positive.
At 5 years: Age 18–39, age ≥60, CCI 1, malignancy grade 3
At 10 years: Breast-conserving surgery, tumor size >20 mm, any chemotherapy (*p* = 0.002).
At both 2 and 5 years: Any adjuvant endocrine therapy.

*BRCAwt* wild-type *BRCA1* and *BRCA2* genes; *BRCApv* pathogenic variants in the BRCA1 and BRCA2 genes; *CI* confidence interval; *CRRM* contralateral risk-reducing mastectomy; *HER2* human epidermal growth factor receptor 2; *HR* hazard ratio; *RRBSO* risk-reducing bilateral salpingo-oophorectomy; *CCI* Charlson Comorbidity Index; *RT* radiotherapy; *TNBC* triple-negative breast cancer, i.e. ER-negative/HER2-negative; *FNA*, fine-needle aspirate.

**Online Resource 3**

**Table 6** Multivariable Cox proportional hazards model for time to death (overall survival) with adjusted HR and 95% CI for overall and time-varying (initial and late) effects

|  |  | **Overall effect**  Adjusted HR, (95% CI) | *p*-value | **Initial effect^a^**  Adjusted HR, (95% CI) | | | **Late effect**  Adjusted HR, (95% CI) | Heterogeneity *p*-value |
| --- | --- | --- | --- | --- | --- | --- | --- | --- |
| ***BRCA* status** | | Ref = *BRCA*wt | 0.08 |  | | |  |  |
|  | *BRCApv* | 1.24 (0.97–1.58) |  | 1.25 (0.99–1.59) | | |  |  |
| **CRRM** | | Ref = No | <0.001 |  | | |  |  |
|  | Yes, time-dependent | 0.64 (0.51–0.81) |  | 0.64 (0.51–0.81) | | |  |  |
| **RRBSO** | | Ref = No | 0.66 |  |  |  | |  |
|  | Yes, time-dependent | 0.96 (0.80–1.16) |  | 0.98 (0.81–1.18) | | |  |  |
| **Age at diagnosis (years)** | | Ref = 40 – 59 | <0.001 |  | | |  |  |
|  | 18–39 | 1.15 (0.97–1.36), |  | 1.31 (1.08–1.57) | | | 0.67 (0.44–1.00) | 0.003 |
|  | ≥60 | 1.93 (1.60–2.34) |  | 1.62 (1.30–2.03) | | | 3.32 (2.44–4.52) | <0.001 |
| **CCI** | | Ref = CCI 0 | <0.001 |  | | |  |  |
|  | CCI 1 | 1.31 (1.04–1.64) |  | 1.07 (0.80–1.41) | | | 2.31 (1.58–3.37) | 0.001 |
|  | CCI 2+ | 1.79 (1.33–2.40) |  | 1.77 (1.32–2.37) | | |  |  |
| **Definitive surgery** | | Ref = Mastectomy -RT | <0.001 |  | | |  |  |
|  | Breast-conserving | 0.64 (0.52–0.78) |  | 0.36 (0.22–0.59) | | | 0.68 (0.55–0.84) | 0.003 |
|  | Mastectomy +RT | 1.00 (0.77–1.29) |  | 1.00 (0.77–1.29) | | |  |  |
| **Histological subtype** | | Ref = Ductal NOS | 0.68 |  | | |  |  |
|  | Lobular | 1.07 (0.84–1.37) |  | 1.11 (0.86–1.42) | | |  |  |
|  | Other or unknown (2 years) | 0.89 (0.63–1.26) |  | 2.53 (1.38–4.62) | | | 1.21 (0.71–2.07) | 0.03 |
|  | Other or unknown (5 years) |  |  |  | | | 0.58 (0.34–0.98) | 0.04 |
| **Malignancy grading** | | Ref = Grade 1 | <0.001 |  | | |  |  |
|  | Grade 2 | 1.27 (1.02–1.59) |  | 1.30 (1.04–1.63) | | |  |  |
|  | Grade 3 | 1.54 (1.20–1.97) |  | 2.17 (1.57–2.99) | | | 1.40 (1.06–1.86) | 0.009 |
| **Tumor diameter** | | Ref = ≤20mm | <0.001 |  | | |  |  |
|  | >20 mm | 1.39 (1.19–1.62) |  | 1.38 (1.19–1.61) | | |  |  |
| **ER/HER2** | | Ref = ER+/HER2– | <0.001 |  | | |  |  |
|  | TNBC | 1.10 (0.85–1.43) |  | 1.08 (0.77–1.52) | | | 0.61 (0.42–0.88) | 0.01 |
|  | HER2+ | 0.66 (0.53–0.82) |  | 0.22 (0.10–0.48) | | | 0.68 (0.54–0.85) | 0.004 |
|  | Unknown | 0.67 (0.50–0.91) |  | 0.58 (0.42–0.79) | | |  |  |
| **Nodal status** | | Ref = Node-negative | <0.001 |  | | |  |  |
|  | 1–3 positive nodes | 1.38 (1.12–1.70) |  | 1.33 (1.08–1.63) | | |  |  |
|  | ≥4 positive nodes/FNA-positive (0–2 years) | 2.68 (2.14–3.36) |  | 4.59 (2.91–7.25) | | | 2.71 (2.10–3.48) | 0.03 |
|  | ≥4 positive nodes/FNA-positive (>10 years) |  |  |  | | | 1.68 (1.16–2.44) | 0.02 |
| **(Neo)adjuvant chemotherapy** | | Ref = Not administered | <0.001 |  | | |  |  |
|  | Any | 0.73 (0.60–0.87) |  | 0.76 (0.63–0.92) | | |  |  |
| **Adjuvant endocrine therapy** | | Ref = Not administered | <0.001 |  | | |  |  |
|  | Any (0–2 years) | 0.61 (0.50–0.74) |  | 0.18 (0.10–0.30) | | | 0.35 (0.25–0.49) | 0.01 |
|  | Any (>5 years) |  |  |  | | | 0.85 (0.67–1.07) | <0.001 |

^a^ Variables showing time-varying effects:
At 2 years: Breast-conserving surgery, HER2+.
At 5 years: Malignancy grade 3, TNBC.
At 10 years: Age 18–39, Age ≥60, CCI 1.
At both 2 and 5 years: histological subtype other/unknown, any adjuvant endocrine therapy.
At both 2 and 10 years: ≥4 pos. nodes/FNA-positive.

*BRCAwt* wild-type *BRCA1* and *BRCA2* genes; *BRCApv* pathogenic variants in the *BRCA1* and *BRCA2* genes; *CI* confidence interval; *CRRM* contralateral risk-reducing mastectomy; *HER2* human epidermal growth factor receptor 2; *HR* hazard ratio; *RRBSO* risk-reducing bilateral salpingo-oophorectomy; *CCI* Charlson Comorbidity Index; *RT* radiotherapy; *TNBC* triple-negative breast cancer, i.e. ER-negative/HER2-negative; *FNA* fine-needle aspirate.
